# Intracolonic migration and anal protrusion of the abdominal catheter of a Ventriculoperitoneal shunt in a patient with diverticulosis: a case report

**DOI:** 10.1093/omcr/omaf214

**Published:** 2025-10-29

**Authors:** Niels Nordin, Mamoun Ahmed, Frederik Boxberg, Semen Semerikov, Martin Scholz, Suzin Jung, Robert Lucaciu

**Affiliations:** Department of Neurosurgery, SANA Hospital Duisburg, Academic Teaching Hospital of the University Duisburg-Essen, Zu den Rehwiesen, 9, 47055, Duisburg, Nordrhein-Westfalen, Germany; Department of Neurosurgery, SANA Hospital Duisburg, Academic Teaching Hospital of the University Duisburg-Essen, Zu den Rehwiesen, 9, 47055, Duisburg, Nordrhein-Westfalen, Germany; Department of Neuroradiology, SANA Hospital Duisburg, Zu den Rehwiesen, 9, 47055, Duisburg, Nordrhein-Westfalen, Germany; Department of Neurosurgery, SANA Hospital Duisburg, Academic Teaching Hospital of the University Duisburg-Essen, Zu den Rehwiesen, 9, 47055, Duisburg, Nordrhein-Westfalen, Germany; Department of Neurosurgery, SANA Hospital Duisburg, Academic Teaching Hospital of the University Duisburg-Essen, Zu den Rehwiesen, 9, 47055, Duisburg, Nordrhein-Westfalen, Germany; Department of Neurosurgery, SANA Hospital Duisburg, Academic Teaching Hospital of the University Duisburg-Essen, Zu den Rehwiesen, 9, 47055, Duisburg, Nordrhein-Westfalen, Germany; Department of Neurosurgery, SANA Hospital Duisburg, Academic Teaching Hospital of the University Duisburg-Essen, Zu den Rehwiesen, 9, 47055, Duisburg, Nordrhein-Westfalen, Germany

**Keywords:** Ventriculoperitoneal, shunt, diverticulosis, anal, protrusion, case report

## Abstract

Ventriculoperitoneal (VP) shunt migration and anal protrusion is a very rare complication of VP Shunt Implantation, which is more often seen in children then adults. We present a case of a 72-year-old female with severe diverticulosis and anal protrusion of VP shunt, 2 years after placement. She presented herself in our emergency room with chronic abdominal pain. Upon further examination, we observed a transanal distal catheter protrusion. Due to the imminent risk of developing meningitis, we opted for emergent removal of the VP Shunt. Simultaneously, through an explorative laparoscopy, the visceral surgeon removed the distal VP shunt catheter, which entered the sigmoid colon through a ruptured diverticulum. This case highlights the importance of recognizing and managing rare complications of VP shunts, particularly in elderly patients with diverticulosis. Early detection and appropriate surgical intervention are crucial in preventing severe consequences such as meningitis and sepsis, as well as ensuring a successful outcome.

## Introduction

Ventriculoperitoneal (VP) shunt placement is a widespread neurosurgical procedure in patients with hydrocephalus. Complication rates remain high, with failure rates up to 11–25% in the first year after the initial placement. Complication rates are more common in the pediatric population than in adults [1]. The most common causes of shunt failure are obstruction and mechanical failure, followed by excessive drainage, lobulations of the ventricular system, infections, and abdominal complications. Abdominal pseudocysts are the most prevalent intraabdominal complication, followed by catheter tip blockage and shunt migrations. The latter tends to happen with possible external extrusion from the perioral or anal region. However, it remains exceedingly rare [2]. Bowel perforation could lead to a retrograde translocation of bacteria that could ascend through the device, causing ventriculitis, meningitis, and sepsis. This complication has a high mortality rate of 15% [3]. This, in return, prompts the emergent need for treatment of abdominal VP shunt-related complications. We present a case of intracolonic migration and anal protrusion of the abdominal catheter of a VP shunt in a patient with diverticulosis for educational purposes.

‘This case report has been reported in line with the CARE guidelines for case reports [4]’.

## Case report

A 72-year-old female presented to our emergency room with chronic abdominal pain, accompanied by mild septic findings in her laboratory analysis (Table 1). The patient had no neurological symptoms and reported that there was a catheter protruding through her anus, after she visited the toilet Her medical history included the placement of aVP shunt two years before presentation to treat normal pressure hydrocephalus (NPH). Her medical history also included an open appendectomy as well as a bladder tumor removal surgery. A contrast-enhanced CT scan of the skull, abdomen and pelvis was performed immediately and revealed a correct location of the VP shunt in the right lateral ventricle of the brain as well as the migration of the distal VP shunt catheter through a diverticular pouch in the sigmoid colon without evidence of intra-abdominal abscess formation (Fig. 2A-C). Upon further extensive Physical examination, we observed a transanal distal catheter protrusion (Fig. 2D). An emergency operation was carried out. The neurosurgical team started with the removal of the ventricular catheter, subsequently the neurosurgical team disconnected the distal catheter just below the valve of the VP Shunt. Simultaneously, through an explorative laparoscopy, the visceral surgeon could pull the distal VP shunt catheter in to the abdominal cavity. The distal catheter entered the sigmoid through a ruptured diverticulum (Fig. 2E and F). Then the distal catheter was pulled out by the OR nurse transanally, so that no Bacteria were pulled cranially. Furthermore, multiple intestinal diverticula were observed intraoperatively. The procedure also included peritoneal adhesiolysis, intraperitoneal lavage, and suturing of the Sigmoid perforation (Fig. 2G).

**Figure 1:**
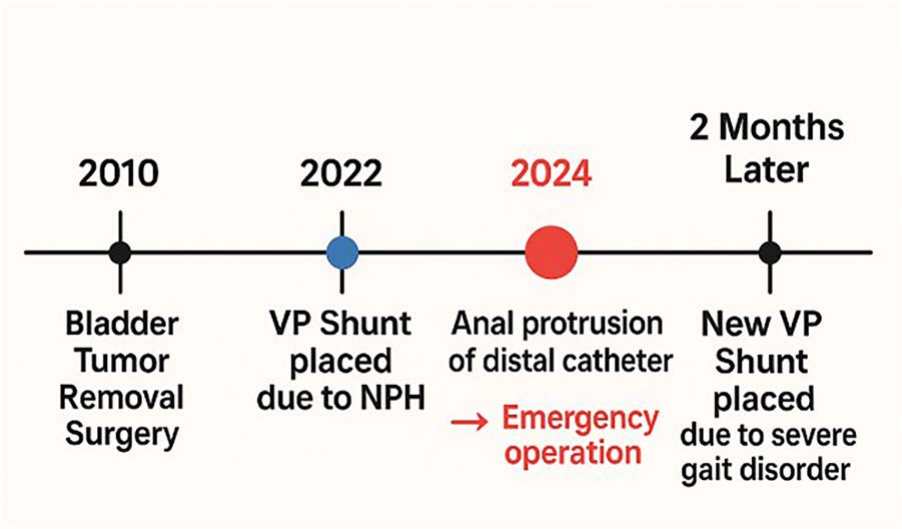
A summarized chronological timeline of the important events.

**Table 1:** Laboratory results at admission.

Lab results	Patient values	Normal values
Leucocytes	8,7/nl	4.0–10.0/nl
Hemoglobin	10,8 g/dl	13.5–17.5 g/dl
CRP^a^	0,6 mg/dl	< 0.5 mg/dl
Procalcitonin	0,17 mg/dl	< 0.10 ng/ml
Sodium	139 mmol/l	135–148 mmol/l
Potassium	4,1 mmol/l	3.5–5.5 mmol/l
Creatinin	0,7 mg/dl	< 1.4 mg/dl
GFR^b^	89 mL/min	> 60 mL/min

^a^CRP: C-reactive protein,

^b^GFR: Glomerular filtration rate.

**Figure 2:**
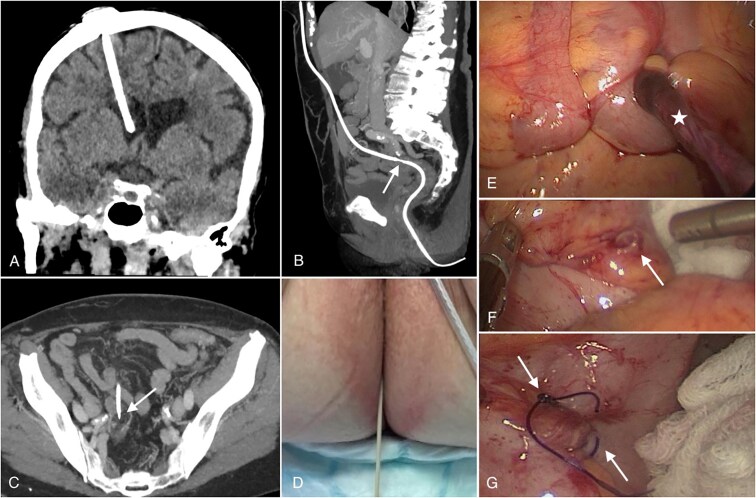
(A): Coronal reconstruction of cranial computed tomography (CT) shows the intraventricular location of the ventriculoperitoneal shunt tip in the frontal horn of the right lateral ventricle. (B): Sagittal maximum intensity projection (MIP) reconstruction of an abdominal CT scan showing the subcutaneous and later intraperitoneal positioning of the shunt before perforating the sigmoid colon (white arrow). (C) Shows the perforation site on an axial abdominal CT scan (marked with a white arrow). (D) Reveals the protrusion of the catheter. (E, F, and G): Laparoscopic pictures showing the catheter (white star in E) as well as the perforation site (diverticulum) in the sigmoid before (arrow in F) and after removal and surgical repair of the perforation site (arrow in G).

On the other hand, the nature of her NPH made emergent external ventricular drainage unnecessary. Postoperatively, the patient was admitted to the intensive care unit (ICU), and broad-spectrum antibiotics (Piperacillin 4 g, Tazobactam 0,5 g) was administered 4 times daily with prolonged administration over 3 hours for 4 Days. Then blood cultures identified *Staphylococcus epidermidis*, prompting adjustment of antibiotics to flucloxacillin 2 g 6 times daily for 10 days according to sensitivity and resistance profiles. Further microbiological investigation of cerebrospinal fluid (CSF) and the ventricular catheter showed no signs of colonization. Laboratory Analysisand CSF analysis following a two-week course of antibiotics revealed no signs of infection. Despite the complications, new placement of a VP shunt was performed due to the development of severe gait disorders in the absence of a VP shunt. The surgery was performed with distal catheter placement under laparoscopic vision. The patient was discharged and reported clinically well at the four-week follow-up, and there were no complications. To illustrate the handling and resolution of a rare VP-shunt-related complication, this case study is provided for educational purposes.

## Discussion

We present a case of a patient with intracolonic migration of a distal VP shunt catheter accompanied by its anal protrusion. Bowel perforation in patients with VP shunts is estimated to occur between 0.1–0.7%. Among the gastrointestinal (GI) tract sites involved, the colon is the most commonly affected (56%), followed by the stomach (28%), with the small intestine being the least common site (16%) [2]. The prevailing hypothesis behind catheter migration suggests the formation of fibrous tissue between the catheter tip and abdominal organs. This fibrous tissue, combined with the continuous pressure of the CSF, could soften the intestinal wall, leading to perforation and invagination of the catheter. The absence of abdominal symptoms or radiological findings may be explained by the fibrosis that has formed around the perforation site, creating a seal that keeps air or bowel contents from entering the peritoneal cavity [1].

Allouh et al. categorized three anatomical migration patterns. Type I (internal migration) involves a catheter puncturing the visceral wall without externalization. Type II (external migration) happens when the catheter partially or subcutaneously penetrates the body wall. On the other hand, type III (compound migration) is distinguished by the catheter’s penetration into the visceral lumen and its eventual passage to a natural body opening that extends to the mouth, urethra, vagina, or anus [5]. According to the adopted classification, we could indicate a type III perforation in our case report.

Park et al. revealed that bowel perforation typically develops 18.7 months following the implantation of a VP shunt [6]. Opposed to being an acute occurrence, this suggests that perforation and migration into the colon are chronic processes, which explains the complication’s late presentation in our case study. Studies imply that perforations that occur within three months of shunt placement are more likely to be due to direct injury during distal catheter placement. The prolonged duration between shunt insertion and bowel perforation supports the chronic nature of this process [7].

Most cases in the literature involve pediatric patients; however, few reports describe this complication in adults with severe diverticulosis. The rationale for managing catheter migration involves addressing the compromised catheter and resolving the visceral perforation with the assistance of an abdominal surgeon, which can be removed via transanal extraction, laparoscopy, or laparotomy [3, 8].

The ideal management, as suggested by most authors, involves disconnecting the distal end of the catheter and removing it. This can be done blindly by pulling the shunt from the extrusion site (transanal) or under endoscopic guidance. In our case, we opted for a laparoscopic approach, due to the ease with which we could pull the distal catheter out of the extrusion site, and simultaneously be able to treat the defect region and possible accompanying complications, such as intraabdominal abscess [6].

This case highlights the importance of recognizing and managing rare complications of VP shunts, particularly in patients with diverticulosis. Early detection and appropriate surgical intervention are crucial in preventing severe consequences such as sepsis and ensuring a successful outcome.

## Conclusion

The majority of reported cases of VP-shunt-related complications involve pediatric patients, with few reports describing this issue in adults with severe diverticulosis. This case highlights the necessity for early detection through ultrasound examinations and long-term surveillance of such patients after shunt placement to ensure early detection and management of complications. Further multicentric studies are required to identify predicting factors associated with abdominal complications related to VP shunts, thereby providing more comprehensive evidence and improving patient outcomes.

## Patient Perspective

Overall patient satisfaction was high because action was taken so quickly. No complications also occurred postoperatively.
